# Health professionals' advice for breastfeeding problems: Not good enough!

**DOI:** 10.1186/1746-4358-3-22

**Published:** 2008-09-11

**Authors:** Lisa H Amir, Jennifer Ingram

**Affiliations:** 1 Mother & Child Health Research, La Trobe University, Melbourne, Australia; 2 Key Centre for Women's Health in Society, University of Melbourne, Melbourne, Australia; 3 Centre for Child & Adolescent Health, University of Bristol, Bristol, UK

## Abstract

Jane Scott and colleagues have recently published a paper in the *International Breastfeeding Journal *showing that health professionals are still giving harmful advice to women with mastitis. We see the management of mastitis as an illustration of health professionals' management of wider breastfeeding issues. If health professionals don't know how to manage this common problem, how can they be expected to manage less common conditions such as a breast abscess or nipple/breast candidiasis? There is an urgent need for more clinical research into breastfeeding problems and to improve the education of health professionals to enable them to promote breastfeeding and support breastfeeding women.

## Editorial

*"Acute mastitis is an all too common disease which has not had the attention it deserves" *[1] (p. 635).

Mastitis is "an inflammatory condition of the breast, which may or may not be accompanied by infection" [2] (p. 1). Scott et al's paper recently published in the *International Breastfeeding Journal *shows that health professionals are still giving harmful advice to women with mastitis [3]. Ten percent of women were advised to stop breastfeeding and many were prescribed an inappropriate antibiotic [3]. In practice, we regularly hear stories from women with mastitis about incorrect advice they have been given by their health care providers: overuse of antibiotics, misuse of antibiotics (wrong medicine or wrong dose), advice to stop breastfeeding (either because of the mastitis or "concerns" regarding the effect of maternal medicines on the infant), or misplaced emphasis on maternal rest leading to skipping feeds overnight.

Mastitis can be seen as an illustration of health professionals' management of wider breastfeeding issues. Mastitis is a problem experienced by 15 to 20% of breastfeeding women [3-5]; women find it distressing, both physically and emotionally [6,7]. Since it is not always caused by an infection, but may be the result of poor milk drainage, it may not require antibiotics (see Breastfeeding Network leaflet for self-help measures [8]). If health professionals don't know how to manage this common problem, how can they be expected to manage less common conditions such as a breast abscess or nipple/breast candidiasis?

Mastitis is poorly researched:

- compared to breastfeeding in general, there have been few papers on mastitis; a rough estimate using PubMed to search for "mastitis (limited to humans)" and for "breastfeeding" reveals 45 publications about mastitis and 247 about breastfeeding in 1977 (1:5.5) – 30 years later in 2007, there were 81 publications on mastitis and 1386 on breastfeeding (1:17.1);

- there is no agreed definition or diagnostic criteria [9];

- there are few clinical treatment trials [10,11];

- and there are few studies on the effects of mastitis and its treatment on infant health [12].

Nipple/breast candidiasis is even less well recognised, managed and researched, and if not treated early often causes women to stop breastfeeding [13]. The range of symptoms are often masked by trauma due to poor positioning and attachment; and treatment compromised if only mother or baby are treated rather than both of them [14].

Renfrew and colleagues have called for urgent research into breastfeeding problems, such as nipple pain and mastitis [11]; their review of interventions to promote and support breastfeeding found no studies of maternal problems related to breastfeeding that met their inclusion criteria [15]. Renfrew has also stressed the importance of educating and preparing health professionals to promote breastfeeding and support breastfeeding women [16].

Over the last 20 years, our advice to women with mastitis is almost the same as that given in a handout written by Amir in 1991 [17]. We still don't have evidence to show whether heat or cold is more effective, when and for how long antibiotics are needed, which is the preferred antibiotic, or when investigations should be undertaken. In recent years, other areas of women's health have received attention from researchers and clinicians, but breastfeeding problems continue to be neglected – or perhaps "invisible" [18].

Felicity Savage recognised that mastitis required the attention of the World Health Organization (WHO), and commissioned the review for WHO that was published in 2000 [2]. However, the findings of the review have not been translated into practice by many health professionals – even if clinicians were aware of the review, just providing information is not enough to change practice [19].

One day, can we hope to see an international meeting on these topics along the lines of the Bellagio consensus meeting on lactational amenorrhea [20]? Meeting by an Italian lake would be idyllic, but we're willing to meet anywhere to make a start on giving breastfeeding problems the attention they deserve.

## Competing interests

The authors declare that they have no competing interests.

## Authors' contributions

LHA and JI co-wrote the paper.
